# Development of bioinks for 3D printing microporous, sintered calcium phosphate scaffolds

**DOI:** 10.1007/s10856-021-06569-9

**Published:** 2021-08-14

**Authors:** Sergio A. Montelongo, Gennifer Chiou, Joo L. Ong, Rena Bizios, Teja Guda

**Affiliations:** Department of Biomedical Engineering and Chemical Engineering, The University of Texas at San Antonio, San Antonio, TX 78249 USA

## Abstract

Beta-tricalcium phosphate (β-TCP)-based bioinks were developed to support direct-ink 3D printing-based manufacturing of macroporous scaffolds. Binding of the gelatin:β-TCP ink compositions was optimized by adding carboxymethylcellulose (CMC) to maximize the β-TCP content while maintaining printability. Post-sintering, the gelatin:β-TCP:CMC inks resulted in uniform grain size, uniform shrinkage of the printed structure, and included microporosity within the ceramic. The mechanical properties of the inks improved with increasing β-TCP content. The gelatin:β-TCP:CMC ink (25:75 gelatin:β-TCP and 3% CMC) optimized for mechanical strength was used to 3D print several architectures of macroporous scaffolds by varying the print nozzle tip diameter and pore spacing during the 3D printing process (compressive strength of 13.1 ± 2.51 MPa and elastic modulus of 696 ± 108 MPa was achieved). The sintered, macroporous β-TCP scaffolds demonstrated both high porosity and pore size but retained mechanical strength and stiffness compared to macroporous, calcium phosphate ceramic scaffolds manufactured using alternative methods. The high interconnected porosity (45–60%) and fluid conductance (between 1.04 ×10^−9^ and 2.27 × 10^−9^ m^4^s/kg) of the β-TCP scaffolds tested, and the ability to finely tune the architecture using 3D printing, resulted in the development of novel bioink formulations and made available a versatile manufacturing process with broad applicability in producing substrates suitable for biomedical applications.

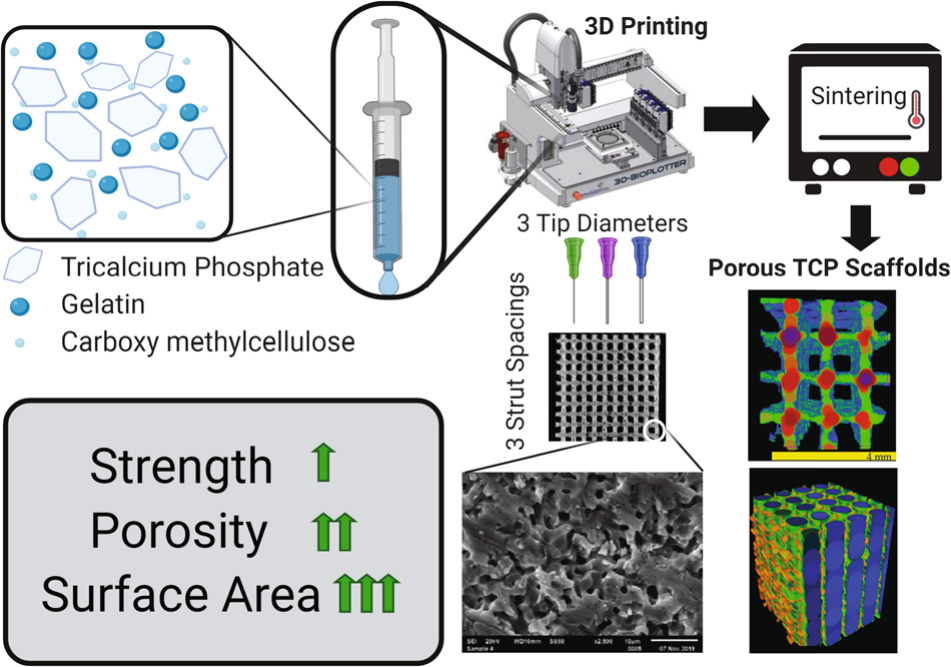

## Introduction

Ceramics, such as synthetic calcium phosphates, have attracted the interest of biomedical researchers and have been used in implant applications in bone [1]. In such case, the material choice proved judicious because calcium phosphates have similar chemical composition and structure as native bone and are, thus, biocompatible. The advent of tissue engineering enhanced scientific interest on calcium phosphates (specifically, beta-tricalcium phosphate (β-TCP) [2, 3]) for skeletal and dental/maxillofacial tissues [4], because these materials could be formulated as porous scaffolds and could, upon degradation, release Ca^2+^ ions. The aforementioned characteristics are necessary specifications required for success in tissue engineering applications. In order to obtain new tissue formation (the ultimate objective of all tissue engineering endeavors), the ceramic scaffolds must provide appropriate architecture (e.g., open and highly porous structures) and appropriate chemical cues (e.g., select ions and growth factors that promote cell functions pertinent to new tissue formation), and scaffold-material degradation synchronous with the rate of new tissue formation [5, 6]. In the case of bone-related applications, the dynamic milieu of that tissue requires that the ceramic scaffolds must also have appropriate mechanical properties; in this respect, ceramics have limitations since they undergo brittle failure under compressive loading.

The aforementioned list of requirements for successful use of calcium phosphates for biomedical applications first required development of appropriate design and processing methodologies. To date, several processing methods have been investigated in order to produce porous, sintered, calcium phosphate architectures [7] including freeze drying [8] and polymer foam templating [9], which sometimes used porogens [3] and molds [10] in combination with calcium phosphate slurries. More recently, additive manufacturing or 3D printing [11–13] enabled greater architectural control and incorporation of desirable macroporosity in material structures. Specifically, 3D plotting, selective laser sintering, stereolithography, direct ink writing, and robocasting are all suitable 3D printing techniques for producing 3D calcium phosphate-based materials [4]. Calcium phosphate inks were typically used in conjunction with binders; in some cases, additional porogens were used to obtain internal porosity throughout the ceramic structure. Post-treatments included either debinding treatments of the green body prior to sintering or sintering at elevated temperatures resulting in burning-out of the additives and sintering of the ceramic grains to form cohesive structures.

The main appeal of additive manufacturing processes stems from the ability to offer customizability in scaffold geometries and architectures. Previous calcium phosphate materials and composite blends developed were flowable, cement-based formulations [14] that cured in situ, and this platform technology has been developed to function as extrusion based settable inks for additive manufacturing applications. Since scaffolds created using this technique have the ability to create hierarchal structures within a single structure to create the ideal macropore sizes (without depending on prefabricated templates, sacrificial porogens, or other manufacturing limitations [1]) and leverage cellular responses to pore architecture to then expedite attachment and proliferation. While techniques such as directional freeze-casting have been previously employed for porogen-free and sintering-free approaches, they are limited in terms of pore size and mechanical strength, usually limited to microporosity and directionally anisotropic mechanical properties [15–17]. Additionally, additive manufacturing processes are amenable to the base calcium phosphate particle being structurally altered [18] or doped with additional bioactive ions, to improve osteo-inductivity [19] or mechanical strength [20] as well as to being combined with synergistic manufacturing processes such as freeze-casting [21] to avail benefits of both. Long term, additive manufacturing will have the ability to develop patient specific solutions to fit pre-imaged defect sites. This is especially beneficial since there have been numerous reports that anatomically oriented pore channels [22], and site-specific tunable porosity and permeability [23] have improved tissue regenerative outcomes. Additive manufacturing technology thus requires an optimized set of “inks” which can hold dimensional fidelity [24–27] during printing and then retain structural and architectural features and mechanical strength post-processing. While these processes are associated with higher costs compared to traditional methods of manufacturing scaffolds for biomedical applications, the significant improvements and investment in additive manufacturing process development might result in more comparable economies of scale over time.

The present study advances the applications of β-TCP materials in 3D printing application by addressing some of the limitations of macroporous ceramic biomaterials. This outcome is achieved by designing novel ceramic ink formulations in order to maximize the ceramic phase of the ink with minimal binder addition. Formulations of β-TCP in conjunction with gelatin and carboxymethylcellulose (CMC) as binders were developed for the 3D printing process and tested in order to optimize handleability and sintering to maintain cohesive structure within a macroporous architecture. The overall objective of this study was to achieve sintered, β-TCP scaffolds with a focus on both high compressive strength and high porosity which are requirements for successful biomedical applications in tissue engineering. The novel methodology provided a combination of macroporosity in 3D architecture and microporosity within the ceramic material tested.

## Materials and methods

### Bioink fabrication

Eighteen composite bioinks containing β-TCP (OssGen, Korea) were developed. The first nine (TCP 1–9) bioinks consisted of nine different mass percentages (ranging from 50 to 90%) of ß-TCP and complementary masses of gelatin (MP Biomedicals, Irvine CA). The second set of nine bioinks (TCP + CMC) consisted of the most successful (as measured by printability, handleability, and sinterability) mass percentages of ß-TCP and gelatin from the original TCP bionks but, in addition, they included increasing mass percentages (specifically, 1, 3, and 5%) of CMC (Spectrum Chemical, Gardena, CA). Details of the slurry compositions prepared and used in the present study are shown in Table 1.

**Table 1:** Summary of the bioink compositions and the selection criteria used in the present study

Bioink	ß-TCP (%)	Gelatin (%)	CMC (% of ß-TCP)	Selection criteria		
				Printable	Handleable	Sinterable
Original formulations						
50 TCP	50	50	–	N	–	–
55 TCP	55	45	–	Y	Y	N
60 TCP	60	40	–	Y	Y	N
65 TCP	65	35	–	Y	Y	N
70 TCP	70	30	–	Y	Y	Y
75 TCP	75	25	–	Y	Y	Y
80 TCP	80	20	–	Y	N	–
85 TCP	85	15	–	Y	N	–
90 TCP	90	10	–	Y	N	–
New formulations						
65 TCP + 1 CMC	65	35	1	Y	Y	N
65 TCP + 3 CMC	65	35	3	Y	Y	Y
65 TCP + 5 CMC	65	35	5	N	–	–
70 TCP + 1 CMC	70	30	1	Y	Y	Y
70 TCP + 3 CMC	70	30	3	Y	Y	Y
70 TCP + 5 CMC	70	30	5	N	–	–
75 TCP + 1 CMC	75	25	1	Y	Y	Y
75 TCP + 3 CMC	75	25	3	Y	Y	Y
75 TCP + 5 CMC	75	25	5	N	–	–

The initial bioinks did not include carboxymethylcellulose (CMC), only the later formulations did. The new formulations were based on the most successful original compositions (i.e., those which allowed for printing, handling, and sintering)

*Y* successful, *N* not successful, *“–”* not tested because the bioink was either not printable or not handleable

Briefly, 10 g of ß-TCP (OssGen, Korea) and their corresponding mass of gelatin/ß-TCP (10–50%; w/w) were dissolved in 10 ml of water and 75% polyethylene glycol/ß-TCP (w/w) (PEG, MW = 200; Texas Lab Supply, Lubbock, TX). Either 1, 3, or 5% of CMC/ß-TCP (w/w) was then added. The resulting slurry was kept at 120 °C until a liquid:powder ratio of 0.45 was achieved before rapid cooling in a −80 °C freezer (to prevent further dehydration). The rapid cooling allowed for thermosetting the ink. Each bioink was sequentially loaded at room temperature in 30 ml syringes for 3D printing use.

### 3D printing of green body scaffolds

Each bioink of interest to the present study was loaded into an EnvisionTEC Bioplotter (Gladbeck, Germany) and heated to 37 °C for 30 min (to allow for temperature equilibration prior to printing). The scaffold 3D files were designed using Autodesk Fusion 360 (San Rafael, CA); the stereolithography files were then imported into the Bioplotter RP (Version 3.0.713.1406, EnvisionTEC). Each 3D model was sliced using Bioplotter RP to the desired slice thickness based on the print nozzle used (specifically, 160, 200, and 320 µm slice thickness corresponding to the 200, 250, and 400 µm tip diameter nozzle, respectively). Once sliced, the 3D models were imported to Visual Machines (Version 2.8.115; EnvisionTEC) to be printed. All scaffolds were printed at a bioink temperature of 37 °C, 0.5–0.7 bar pressure, and at a speed of 10–12 mm/s (actual speed based on the bioink composition). A 0.3 s preflow and 0.1 s post-flow were implemented, with the printing nozzle cleaned after every three printed layers. The printing stage was kept at a temperature of 27 °C. Upon completion of the printing process, each printed scaffold was allowed to thermally set at room temperature for 5 min before removal from the printing stage. After a period of at least 72 h, the 3D-printed scaffolds were sintered using a Hot Spot 110 furnace (Zircar Zirconia, Florida, NY) to 1240 °C for a cycle of 20 h.

### Thermogravimetric analysis

Thermogravimetric analysis was performed to determine whether the differences in content of the TCP-CMC bioinks would warrant different sintering temperature profiles. For this purpose, small (20 mg) green body samples (*n* = 4 per ink and averages evaluated) from each of the TCP-CMC bioinks (prepared as described in the “Bioink fabrication” section) were loaded onto a Pyris 1 Thermogravimetric Analyzer (PerkinElmer, Waltham, MA). Each sample was brought up to 600 °C at a ramp rate of 3 °C/min, followed by a dwell at 600 °C for 1 h. The mass of each sample was recorded at a rate of 10 Hz.

### Mechanical testing

Mechanical compression testing was performed on 3D-printed cylinders (*d* = 12 mm, *h* = 24 mm; *n* = 10) using an MTS Insight 5 (MTS systems, Eden Prairie, MN) testing frame equipped with a 5 kN load cell at a compression rate of 1.25 mm/min. This testing was conducted for all nine combinations of slice thickness and tip diameter nozzle described in the “3D printing of green body scaffolds” section. From the stress/strain curve data thus collected, the ultimate stress at failure, strain at failure, toughness and elastic modulus were calculated for all samples tested. In addition, three-point bending tests were performed on rectangular prisms (each of size 10 × 2 × 1.5 mm^3^; *n* = 10) cast within 3D-printed acrylonitrile butadiene styrene molds. This testing was performed for the three bioink formulations with 3% CMC that were printable. The rectangular prims were sintered and tested using a Mach1 testing frame (Biomomentum Inc, Quebec, Canada) at a crosshead velocity of 1.25 mm/min over a span of 6 mm. From the stress/strain data, the flexural moduli for the different sintered bioinks were calculated.

### Porosity characterization

Porosity measurements were determined from the solid volume fraction of the 3D-printed scaffolds of interest to the present study. The skeletal volume for each cylindrical sample was measured six times using an AccuPyc II helium pycnometer (Micromeritics, Norcross, GA). The height and diameter of each cylinder tested were measured at three independent times and, subsequently, the data were averaged; these measurements were used to calculate the envelope volume of each cylinder. The porosity of each sample of interest to the present study was then determined by the ratio of the skeletal volume to the envelope volume obtained from the geometric dimensions of each sample according to Eq. (1):

1 $${{{{{\mathrm{Percent}}}}}}({{{{{\mathrm{\% }}}}}}){{{{{\mathrm{Porosity}}}}}} = \left( {1 - \frac{{{{{{{\mathrm{Skeletal}}}}}}\,{{{{{\mathrm{Volume}}}}}}}}{{{{{{{\mathrm{Envelope}}}}}}\,{{{{{\mathrm{Volume}}}}}}}}} \right) \times 100{{{{{\mathrm{\% }}}}}}$$

All scaffolds were weighed, and the specific gravity of the ß-TCP component was used as a control to verify the accuracy of the pycnometry measurements.

### Microcomputed tomography (µCT) analysis

µCT scans of each 3D-printed cylindrical samples was performed using the SkyScan 1076 (Bruker, Kontich, Belgium) at 8.87 μm spatial resolution, and reconstructed using nRecon64 v.1.7.3 (Bruker µCT, Kontich, Belgium). Morphometric analysis was then carried out on the µCT images using CTanalyzer v.1.17.7.2 (Buker, Kontich, Belgium). In order to determine the scaffold architecture, 12 traditional histomorphometric parameters were computed for each of the tested samples over a cubic volume of interest (specifically, 4.05 × 4.05 × 4.05 mm^3^) at the midsection. The 11 parameters computed were the following: scaffold volume ratio (SV/TV), scaffold surface to scaffold volume ratio (SS/SV), scaffold surface density (SS/TV), strut pattern factor (StPf), structural model index (SMI), strut thickness (StTh), strut number (StN), strut spacing (StSp), degree of anisotropy (DA), closed porosity, and connectivity.

### Scanning electron microscopy (SEM)

3D-printed, sintered ß-TCP rectangular prisms (each one 12 × 12 × 1.5 mm^3^) were polished using increasing (specifically, 320, 400, 600, and 1000) grit silicon carbide paper each at 200 rpm for 1 min. The polished scaffolds were characterized for morphology following sputter coating with gold and palladium (STEM Hitachi S5500). SEM imaging was obtained using a JEOL JSM-6610LV Scanning Electron Microscope (JEOL Ltd, Akishima, Tokyo, Japan) at an applied voltage of 20 kV and a magnification factor of up to ×2500.

### X-ray diffraction (XRD) analysis

The crystallinity of the TCP-CMC ceramic bioink after sintering was determined using XRD analysis. For this purpose, each sintered sample was ground to a fine powder, loaded onto a glass holder, and analyzed using an Ultima IV X-ray diffraction system (Rigaku, Tokyo, Japan). Pertinent spectra were collected from 20° to 60° (2θ) at a resolution of 0.02°.

### Scaffold inner architecture variation

Rectangular prims (each 12 × 12 × 1.5 mm^3^) and cylinders (12 mm diameter and 24 mm height; *n* = 8) were printed using a 75%-TCP + CMC bioink. This bioink composition was selected because of its superior mechanical performance under compression compared to the 65 and 70% TCP + CMC bioinks. The inner pattern of each printed scaffold was set as linear print nozzle movement with 90º rotation between layers and printed without contours. A total of nine different architectures were designed by varying the print spacing (specifically, 1.2, 1.4, and 1.8 mm) and conical tip diameters (specifically, 200, 250, and 400 µm).

### Pore and strut spatial analysis

The strut thickness and pore spacing of the green body and sintered rectangular prism scaffolds (described in the “Scaffold inner architecture variation” section) were measured from micrographs obtained using a Leica DM IL LED light microscope (Leica, Wetzlar, Germany). Three randomly-selected, on-focus struts and pores were measured at a magnification of ×10 in three different scaffolds using the Bioquant Osteo program (v10.3.6, Bioquant, Nashville, TN). The same scaffolds were measured in their green body and sintered body states and averages were obtained for each architecture of the two states.

### 3D scaffold permeability determination

The ß-TCP scaffold permeability was determined using a custom flow apparatus consisting of an open to the atmosphere fluid reservoir that fed into each cylindrical scaffold (*n* = 10) tested. The pressure head was, thus, equal to the height of the liquid column above the sample chamber. Using Darcy’s law, the liquid permeability of each scaffold tested was determined using Eq. (2):

2 $${{k}} = \frac{{{{m}}\mu {{L}}}}{{{{A}}_{{{{{{\mathrm{CS}}}}}}}\rho {{{{{\mathrm{{\Delta}}}}}P}}}}$$

where *k* is the liquid permeability for each tested sample (m^2^), *m* is the mass flow rate, *µ* is the fluid viscosity, *L* is the scaffold height, *A*_cs_ is the mean cross-sectional area of the tested sample, *ρ* is the fluid density, and ∆*P* is the pressure drop across the scaffold tested. Deionized water was used for the permeability measurements. Each scaffold was allowed to equilibrate by soaking in deionized water at room temperature for at least 5 min prior to testing. Permeability was then determined by measuring the time required to collect three different volumes of deionized water (specifically, 50, 100, and 200 ml) flowing through each scaffold tested. The flow rate was measured at three different times per tested volume of effluent water, per scaffold. Using Eq. (3), the conductance of each scaffold was determined as:

3 $${{C}} = \frac{{{{dQ}}}}{{{{dP}}}}$$

where *C* is the conductance and *Q* is the relative induced flow through the scaffold per unit pressure drop (*P*).

### In vitro cell proliferation and activity

Scaffolds were sterilized by autoclaving and seeded with primary stem cells from human exfoliated deciduous teeth (SHEDs, passage 4) (iXCells Biotechnologies, San Diego, CA). In total, 1.25 × 10^5^ cells were added per scaffold (12 × 12 × 1 mm^3^), submerged in 1 ml media. SHED Growth Medium was comprised of 20% v/v fetal bovine serum, 1% v.v Antiobiotic-Antimycotic (100x), 1% v/v SHEDs growth supplement (100x) (iXCells), and 0.2% Mycozap (Lonza, Basel, Switzerland). Cells were allowed to attach for 8 h, and media was refreshed. The media then was refreshed every 2 days. Samples were incubated at 37 °C and 5% CO_2_. Cell proliferation was assessed via an Alamar Blue assay (Invitrogen, Carlsbad, CA) at days 1, 7, and 14. Briefly, scaffolds were incubated with 500 µl of fresh media with 10% volume of Alamar Blue. After 4 h of incubation, media was transferred to a new well plate and their fluorescence was evaluated using a spectrophotometer (Synergy 2, Biotek, Winooski, VT) and microplate reader software Gen5 (version 2.05.5, Biotek). The bioactivity of the scaffolds was in terms of maintaining an osteogenic phenotype was quantified by RT-qPCR. Briefly, samples were lysed in 500 µl of TRIzol (Thermo Fisher). RNA was extracted using an RNeasy kit (Qiagen, Germantown, MD). RNA quality and concentration was measured using a Take3 Micro-Volume Plate (Biotek) and spectrophotometer. cDNA was obtained from RNA by using iScript Reverse Transcription Supermix (Bio-Rad, Hercules, CA) and a T100 Thermal Cycler, (Bio-Rad) to a concentration of 50 ng/ml. Samples were amplified with iTaq Universal SYBR Green Supermix (Bio-Rad) and *GAPDH*(5′-3′ fwd: AGCCACATCGCTCAGACAC, 5′-3′ rev: GCCCAATACGACCAAATCC) and RUNX2 (5′-3′ fwd: CGTGGCCTTCAAGGTGGTA, 5′-3′ rev: AGCTCAGCAGAATAATTTTC) (Sigma-Aldrich, St. Louis, MO). Fold changes were calculated for *runx2* relative to *GAPDH* levels at day 1.

### Statistical testing

Results are presented as average ± standard error of the mean. All data sets were tested using Grubbs’ extreme studentized deviate test (GraphPad Software, San Diego, CA) to check for the presence of an outlier, which were removed from the test data if found to be significant (*p* < 0.05). The data were then evaluated for normality using the Shapiro–Wilk test and tested to have equal variance using the Brown–Forsythe test prior to being tested using ANOVA if both prior tests passed (*p* > 0.05) or alternately using the Kruskal–Wallis analysis of variance on ranks if that were not the case (SigmaPlot v13, Systat Software Inc, San Jose, CA). Quantitative data obtained in the formulation of the bioinks, including µCT based scaffold volume percentage and trabecular pattern factor, and compressive elastic modulus were compared using one-way ANOVA (across percentage of β-TCP) to determine statistical significance (*p* < 0.05). Post hoc testing was performed using Tukey’s test. Compressive strength was compared by ANOVA on ranks followed by Dunn’s method for determining post hoc statistical significance between groups (*p* < 0.05). Pore size and strut thickness measurements were compared by three-way ANOVA (across print spacing, tip diameter, and sintering state) with post hoc significance assessed using Tukey’s test (*p* < 0.05). Interactions for multiple testing were assessed at *α* = 0.05 and a power of atleast 0.996. Total porosity, fluid conductance, scaffold volume %, scaffold surface density, connectivity density, and strut spacing across the different architectures printed were compared using two-way ANOVA (across print spacing and tip diameter) and post hoc significance was determined by the Holm–Sidak method (*p* < 0.05). Mechanical properties including strength, ultimate strain, elastic modulus, and toughness across the architectures were compared using two-way ANOVA (across print spacing and tip diameter) and post hoc significance was determined using the Student–Newman–Keuls method (*p* < 0.05). Cell proliferation and gene expression analysis were both compared using three-way ANOVA (across time, tip diameter, and print spacing) with post hoc significance (*p* < 0.05) determined using Tukey’s test. Interactions were tested at *α* = 0.05 and with a power of at least 0.95.

## Results

### Bioink fabrication and 3D printing

Compared to the bioinks with no CMC, addition of 3% CMC in β-TCP (w/w) contributed to increased stability of the resultant bioinks prior to sintering. The 1%-CMC-containing bioink was very similar to the initial bioinks without CMC, while addition of 5% CMC made the resultant bioinks very viscous (Supplementary Fig. S1 shows rheological information) at the 70 kPa print pressure and prevented their use in 3D printing. The selection of the three bioinks based on their printability, handleability, and sinterability were the following: 65% β-TCP plus 3% CMC; 70% β-TCP plus 3% CMC; 75% β-TCP plus 3% CMC (Table 1). These three bioinks were fully characterized by the techniques detailed in the “Materials and methods” section.

### Morphometry of the selected bioinks

**Fig. 1:**
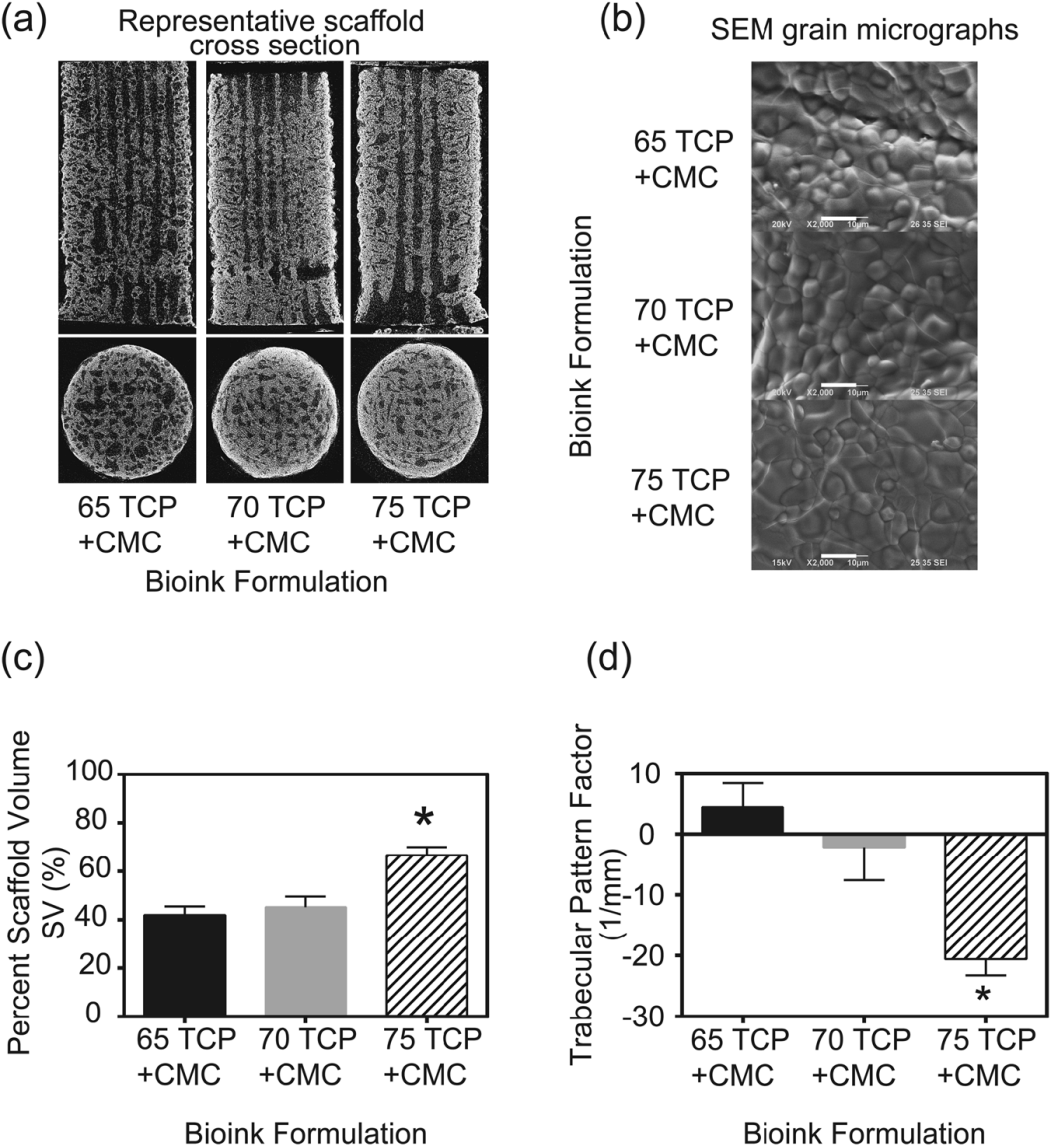
**a** Microcomputed tomography cross-sections of cylinders printed with the three specified formulations of bioinks. **b** SEM micrographs of the cylinders after sintering illustrating similar grain size dimensions for all bioinks. Magnification: ×200. Scale bar = 10 µm. **c**, **d** Morphological parameters demonstrating higher scaffold volume and connectivity post-sintering of the 75 TCP + CMC than the 65 TCP + CMC and 70 TCP + CMC bioinks (*p < 0.05). CMC carboxymethyl cellulose, TCP ß-tricalcium phosphate

**Fig. 2:**
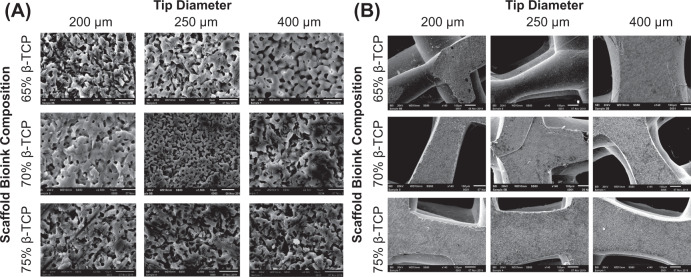
Scanning electron micrographs of scaffolds of three different printed strut thicknesses composed of the three bioink formulations, tested after sintering. **a** High-magnification (×2500) micrographs of the 3D-printed scaffolds structures. The internal microporosity within the struts is clearly distinguishable. **b** Low-magnification (×140) images of the 3D-printed struts of the different scaffolds demonstrating structural reliability

The aforementioned three bioinks were printed and sintered in 3D architectures (Fig. 1a). SEM micrographs of the printed surface revealed that the average grain size of the 65% β-TCP plus 3% CMC, 70% β-TCP plus 3% CMC and 75% β-TCP plus 3% CMC scaffolds were 5.85 ± 0.176 µm, 7.52 ± 0.269 µm, and 6.33 ± 0.171 µm, respectively (Fig. 1b). The results of percent scaffold volume (SV/TV) and strut thickness (StTh) analyses provided evidence that the 75% ß-TCP plus 3% CMC formulation was significantly (*p* < 0.05 for both SV/TV and StTh) different than the other two bioink formulations (specifically, the 65% TCP plus 3% CMC and 70% TCP plus 3% CMC) tested. Higher concentrations of ß-TCP in the bioinks resulted in a higher volume fraction of the ceramic after sintering (higher SV/TV; Fig. 1c), and consequently, resulted in a more connected structure (that is, a more negative TbPf; Fig. 1d). SEM micrographs of the polished strut cross-section showed similar grain sizes across scaffolds printed using various tip diameters for each of the three bioinks tested, and demonstrated both granular fusion as well as distributed intergranular porosity throughout the solid matrix (Fig. 2a). Evaluation of scaffold morphology indicated architectural reliability for all three bioink formulations printed using various tip diameters, with the printed struts holding shape after sintering (Fig. 2b).

### Mechanical strength of the composite bioinks

**Fig. 3:**
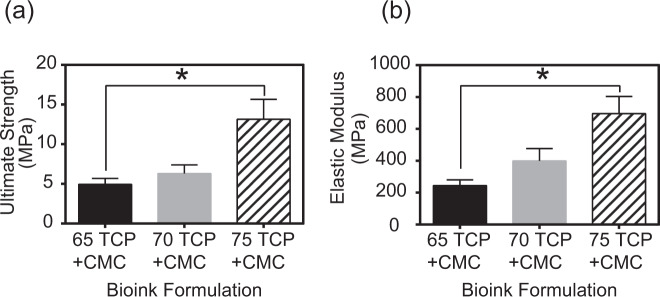
Mechanical properties of the new formulations of the ceramic bioinks after sintering. The ultimate strength (**a**) and modulus (**b**) of the 75% ß-TCP plus 3% CMC composition were highest among the formulations tested. (*p < 0.05). CMC carboxymethyl cellulose, TCP ß-tricalcium phosphate

The compressive testing results provided evidence that the composition of 75% β-TCP plus 3% CMC exhibited significantly (*p* < 0.008) higher ultimate compressive strengths when compared to scaffolds containing 65% β-TCP plus 3% CMC (13.14 ± 2.51 MPa versus 4.94 ± 0.76 MPa, respectively; Fig. 3a). The elastic modulus was also significantly (*p* < 0.05) higher for the scaffolds containing 75% β-TCP plus 3% CMC compared to scaffolds composed of 65% β-TCP plus 3% CMC (696.03 ± 107.78 MPa versus 44.92 ± 35.1 MPa, respectively; Fig. 3b). The ultimate compressive strain (3.8 ± 0.5%) and toughness (126 ± 23 MJ/m^3^) of all bioink compositions tested were similar (data not shown).

### Thermogravimetry and crystal structure

**Fig. 4:**
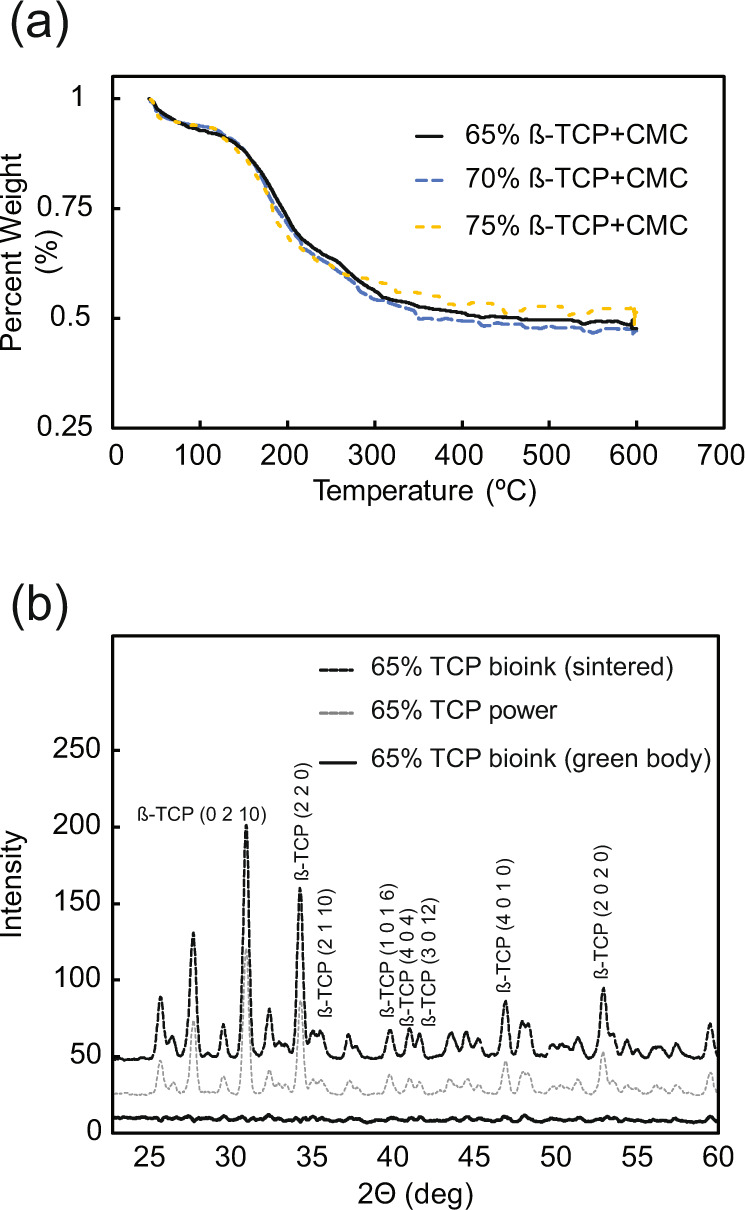
**a** Thermogravimetric analysis of the ß-TCP plus 3% CMC bioinks (green body). All bioinks of interest to the present study exhibited similar loss of mass with respect to temperature due to water and gelatin loss. All bioinks reached an equilibrium at 600 °C. **b** XRD spectra of the 65% TCP plus CMC bioink as green body and after sintering compared to pure TCP powder. CMC carboxymethyl cellulose, TCP ß-tricalcium phosphate

The percent loss of mass exhibited during the thermogravimetric analysis was similar for all three chosen bioinks tested (Fig. 4a). The initial loss of water and gelatin accounted for the observed decrease in the mass of the bioinks before reaching a steady state at 600 °C. The XRD spectrum of pure β-TCP powder (reference peaks from JCPDS #09-0169, ß-TCP) was compared to the spectra of the green body and sintered forms of the ß-TCP plus 3% CMC bioinks. The spectra of the sintered bioinks (Fig. 4b, gray dashed line) representing the 65% TCP + CMC bioink and the pure β-TCP powder (Fig. 4b, black dashed line) were similar, suggesting similar crystalline structure. In contrast, the green body bioink XRD spectrum was mostly flat, typical of amorphous solids (Fig. 4b, black solid line).

### Pores and strut size distribution

**Fig. 5:**
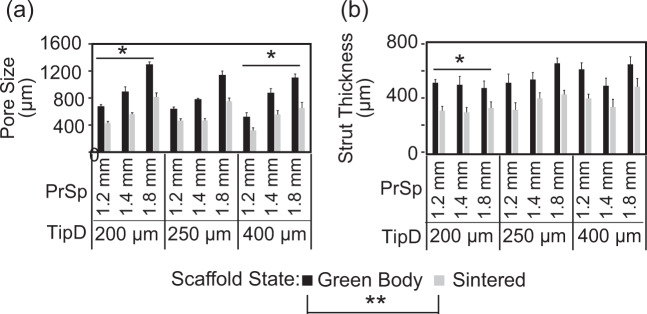
**a** Dimensional analysis of average pore size of the nine 3D-printed scaffold architectures tested in the present study. For all architectures, the sintered samples had a significant (**p < 0.001) reduction in their average pore size. The scaffolds printed using a 200 µm tip diameter showed significantly (*p < 0.05) greater pore size compared to the scaffolds printed with a 400 µm tip diameter. **b** Spatial dimensional analysis of strut thickness in the 3D-printed scaffolds. Sintering significantly (**p < 0.001) reduced the thickness of the struts by ~35%. The struts printed using the 200 µm nozzle were significantly (*p < 0.05) thinner than those printed using either the 250 or 400 µm tip diameters. PrSp print spacing, TipD tip diameter

Scaffolds printed with the 75% β-TCP plus 3% CMC bioink exhibited ~35% shrinkage from their green body form to their final sintered size. This shrinkage rate resulted in statistically (*p* < 0.001) significant smaller pores as well as significantly (*p* < 0.001) thinner struts as compared to the dimensions of the respective green bodies. The sintered pores in the scaffolds printed with 1.2, 1.4, and 1.8 mm print spacing were all significantly (*p* < 0.007) different from each other. Scaffolds with 1.8 mm print spacing had significantly (*p* < 0.02) larger pores within the 200 and 250 µm tip diameter scaffold groups compared to the scaffolds of the 1.2 and 1.4 mm print spacing groups. Among the 400 µm tip diameter group, the 1.2 mm print spacing subgroup exhibited significantly (*p* < 0.02) smaller pore spacing (Fig. 5a). The diameter of the printing tip affected the final thickness of the struts after sintering; the 200 µm tip diameter group had significantly (*p* < 0.05) thinner struts than the 250 and 400 µm tip diameter scaffold groups (Fig. 5b).

### Porosity

**Fig. 6:**
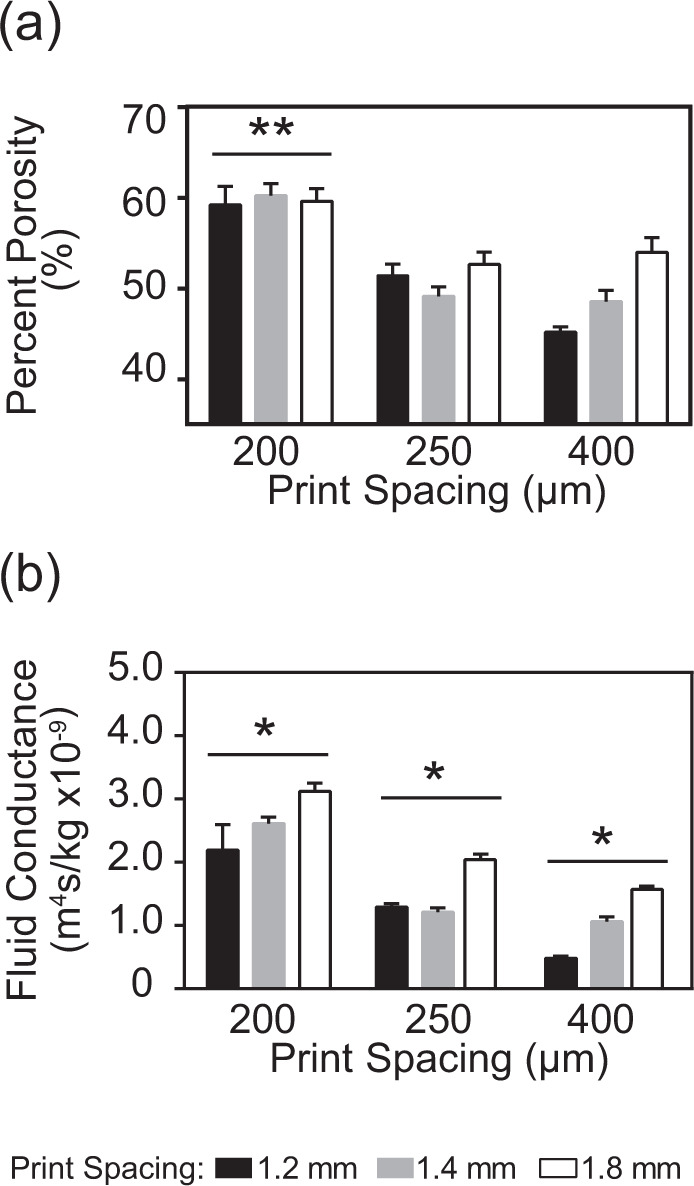
**a** Porosity of the scaffolds printed with varying tip diameters and print spacing. Scaffolds printed with thinner tip diameters (200 µm) exhibited significant (**p < 0.001) increased porosity. **b** Fluid Conductance through the scaffolds printed with various tip diameters and print spacing. The scaffolds printed with different tip diameters displayed significant (*p < 0.02) differences in fluid conductance from each other. The scaffolds printed with the smallest tip diameter (200 µm) and largest print spacing (1.8 mm) displayed the highest conductance. PrSp print spacing, TipD tip diameter

The porosity of the various architectures tested was greater than 45% (range from 45.1–60.2%). Scaffolds printed using a 200 µm tip diameter had a porosity of 59.6 ± 0.09%, a value significantly (*p* < 0.001) higher than that obtained for the scaffolds printed using tip diameters of 250 and 400 µm. The scaffold group printed at a print spacing of 1.8 mm was significantly (*p* < 0.005) more porous (specifically, 55.4 ± 0.01%) than the other two print spacing groups tested in the present study (Fig. 6a).

### Fluid conductance

The calculated water conductance through the 3D-printed scaffolds tested was found to significantly (*p* < 0.02) decrease with increased nozzle tip diameter, specifically 2.66 × 10^−9^ ± 2.10 × 10^−10^, 1.51 × 10^−9^ ± 1.34 × 10^−10^, and 1.04 × 10^−9^ ± 1.24 × 10^−10^ m^4^s/kg for the 200, 250, and 400 µm tip diameters, respectively (Fig. 6b). In addition, the scaffolds printed with 1.8 mm print spacing exhibited significantly (*p* < 0.007) higher conductance (2.27×10^−9^ ± 1.98 × 10^−10^ m^4^s/kg) than the other scaffold groups tested, specifically, 1.61 × 10^−9^ ± 1.89 × 10^−10^ m^4^s/kg and 1.32 × 10^−9^ ± 1.95 × 10^−10^ m^4^s/kg, for the 1.4 and 1.2 mm spacing, respectively.

### Scaffold architectural morphometry

**Fig. 7:**
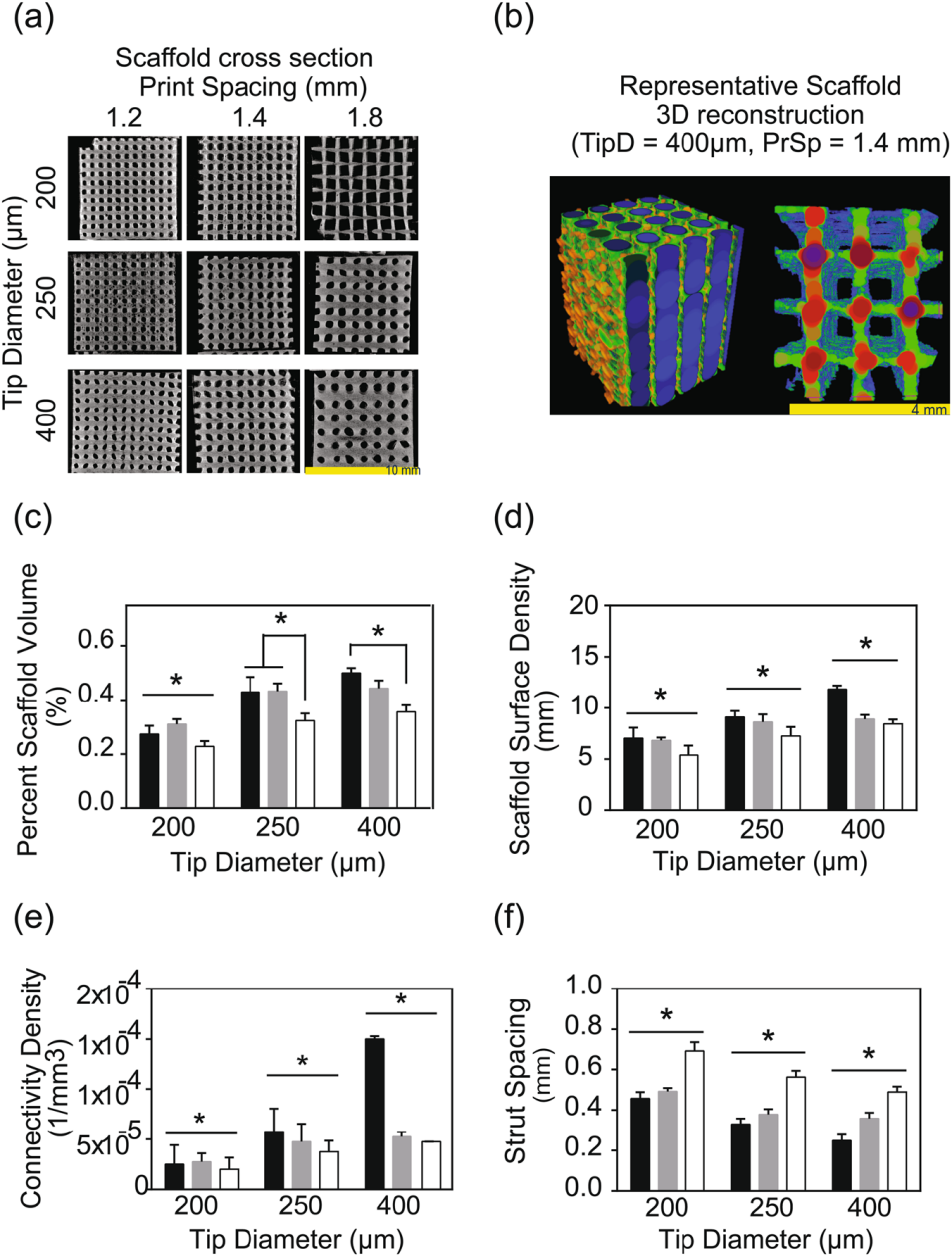
**a** Microcomputed tomography cross-sections of the 75% ß-TCP + CMC scaffolds printed with varying tip diameters and strut spacing. **b** Representative 3D reconstruction of the volume of interest (VOI) of a scaffold with TipD 200 µm, PrSp 1.4 mm, and VOI = 4.05 × 4.05 × 4.05 mm^3^). In the 3D reconstruction, StSp and StTh are displayed in a color map based on sphere filling, specifically, Left: printed pore size distribution where the pore size is uniform and Right: strut thickness uniformity, except for the intersections of perpendicular struts. **c** The percent scaffold volume (SV/TV) increased as TipD increased and decreased as PrSp increased. The 200 µm TipD group had significantly (*p < 0.001) lower SV/TV compared to the other two tip diameter groups tested. Within each TipD group, the SV/TV decreased as the PrSp increased; this trend was significantly (*p < 0.001) different within the scaffolds in the 1.8 mm PrSp group, printed with the 250 and 400 µm tip diameters. **d** The scaffold surface density increased as TipD increased and decreased as PrSp decreased. All TipD tested groups were significantly (*p < 0.001) different from each other. **e** The connectivity density of all TipD groups tested were significantly (**p < 0.05) different from each other. The 400 µm group exhibited the highest and the 200 µm the lowest connectivity density, respectively. The connectivity density also decreased as PrSp increased (**p < 0.05). **f** All TipD groups displayed significantly (*p < 0.001) different strut spacing from each other. The groups printed with 200 µm TipD had the greatest strut spacing and those printed with 400 µm tip diameter displayed the lowest strut spacing. CMC carboxymethyl cellulose, TCP ß-tricalcium phosphate, TipD tip diameter, PrSp print spacing

Cross-sectional µCT slices (Fig. 7a) were reconstructed into volumes of interest for each of the printed scaffolds scanned. From such 3D reconstructions (Fig. 7b), the following morphological parameters were analyzed for all architectural scaffold groups of interest to the present study: percent scaffold volume (SV/TV), scaffold surface density (SS/TV), connectivity density, strut spacing, and strut thickness. The percent skeletal volume of the scaffolds printed with 1.8 mm print spacing (30.29 ± 2.07%) was significantly (*p* < 0.001) lower than the respective values of scaffolds printed with the 1.2 mm (40.16 ± 3.50%) and 1.4 mm (39.62 ± 2.27%) print spacing groups. Furthermore, the scaffold group printed with the 200 µm tip diameter exhibited significantly (*p* < 0.001) lower SV/TV (27.12 ± 1.63%) compared to the pertinent results obtained for the 250 µm (39.57 ± 1.98%) and 400 µm (43.38 ± 2.74) tip diameter groups (Fig. 7c).

In terms of SS/TV, all three tip diameter groups tested in the present study were significantly (*p* < 0.001) different: the group printed with the 400 µm tip diameter had the highest (9.73 ± 0.60 1/mm) and the 200 µm group the lowest (6.42 ± 0.30 1/mm) observed values. The 1.8 mm print spacing displayed significantly (*p* < 0.001) lower SS/TV (7.03 ± 0.56 1/mm) compared to the results (9.33 ± 0.70 1/mm) obtained from the 1.2 mm group (Fig. 7d).

The connectivity density significantly (*p* < 0.04) increased with increasing tip diameter specifically, 2.4 × 10^−5^ ± 1.8 × 10^−5^, 4.8 × 10^−5^ ± 1.8 × 10^−5^, and 8.3 × 10^−5^ ± 1.7 × 10^−5^ for the 200, 250, and 400 µm groups, respectively. In addition, the 1.2 mm print spacing groups exhibited significantly (*p* < 0.007) higher connectivity density (7.8 × 10^−5^ ± 3.7 × 10^−5^) compared to pertinent results obtained for the 1.4 mm (4.3 × 10^−5^ ± 1.4 × 10^−5^) and 1.8 mm print spacing (3.5 × 10^−5^ ± 1.2 × 10^−5^) (Fig. 7e).

Post-sintering strut spacing significantly (*p* < 0.001) decreased with increasing tip diameter, specifically 0.55 ± 0.04 mm, 0.42 ± 0.04 mm, 0.37 ± 0.03 mm for the 200, 250, and 400 µm scaffolds, respectively. Strut spacing also significantly (*p* < 0.001) increased with increased print spacing: 0.35 ± 0.03 mm, 0.41 ± 0.02 mm, and 0.58 ± 0.03 for the 1.2, 1.4, and 1.8 mm print spacing groups, respectively (Fig. 7f). Strut thickness was similar for all print spacing and tip diameter combinations tested in the present study (mean thickness for all groups 0.18 ± 0.01 mm).

### Impact of scaffold architecture on the compressive stress and toughness

**Fig. 8:**
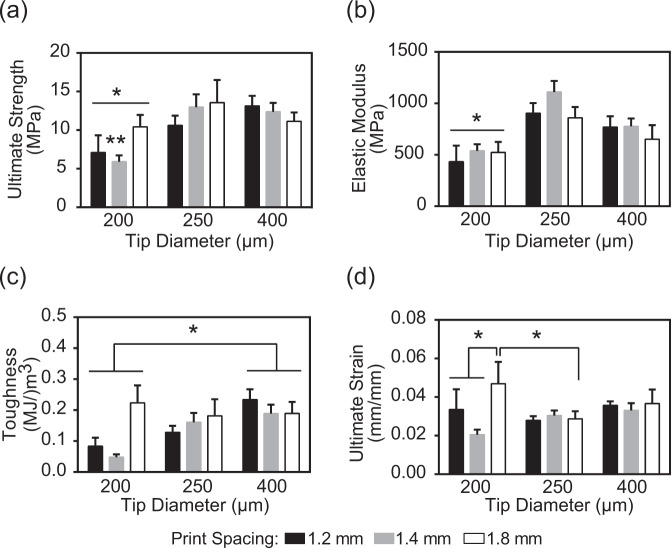
Mechanical properties of the 3D-printed cylindrical scaffolds using various tip diameters and print spacing. **a** The ultimate strength of the scaffolds printed with 200 µm TipD had significantly (*p < 0.05) lower ultimate strength compared to the results obtained from the other two groups tested; the 1.4 mm PrSp exhibited the lowest strength (*p < 0.05) compared to the other two PrSps tested in the present study. **b** The elastic moduli of the scaffolds printed with the 200 µm TipD were significantly (*p < 0.05) lower than those printed with 250 and 400 µm TipD. **c** The toughness of the scaffolds printed with 200 µm TipD was significantly (**p < 0.005) lower than those printed with a TipD of 400 µm. **d** Among the scaffolds printed with a PrSp of 1.8 mm, the ones printed with a TipD of 200 µm had significantly (*p < 0.05) higher ultimate strain than those printed with 250 µm TipD. Within the 200 µm TipD group, the scaffolds printed with a PrSp of 1.8 mm had significantly (*p < 0.05) higher ultimate strain than those printed with a PrSp of either 1.2 or 1.4 mm. TipD tip diameter, PrSp print spacing

The mechanical properties of the scaffolds tested in the present study were affected more by varying the tip diameter than by variations in the print spacing. Specifically, the scaffold groups printed using the 200 µm tip diameter exhibited significantly (*p* < 0.05) lower ultimate compressive strength (7.72 ± 0.96 MPa) and elastic modulus (545.82 ± 72.93 MPa) than the scaffolds printed using the 250 µm (11.12 ± 0.89 MPa and 924.05 ± 66.40 MPa for compressive strength and modulus, respectively) and the 400 µm (11.95 ± 0.77 MPa and 794.56 ± 61.37 MPa for compressive strength and modulus, respectively) tip diameters (Fig. 8a, b). Scaffolds printed with a 200 µm tip also resulted in significantly (*p* < 0.004) lower toughness (0.11 ± 0.027 MJ/m^3^) compared to the results obtained with the 400 µm (0.20 ± 0.019 MJ/m^3^) but not with the 250 µm cohort (0.13 ± 0.017 MJ/m^3^) (Fig. 8c). Scaffolds printed with the 200 µm tip diameter and 1.8 mm print spacing displayed the highest ultimate strain compared to all other architectures tested in the present study; with this strain value being significantly (*p* < 0.02) higher than the ultimate strain for the scaffolds printed using the 1.2 and 1.4 mm print spacing and 200 µm tip diameter as well as from the ultimate strain for the scaffolds printed using the 1.8 mm print spacing and 250 µm tip diameter (Fig. 8d).

### Cell proliferation and gene expression

**Fig. 9:**
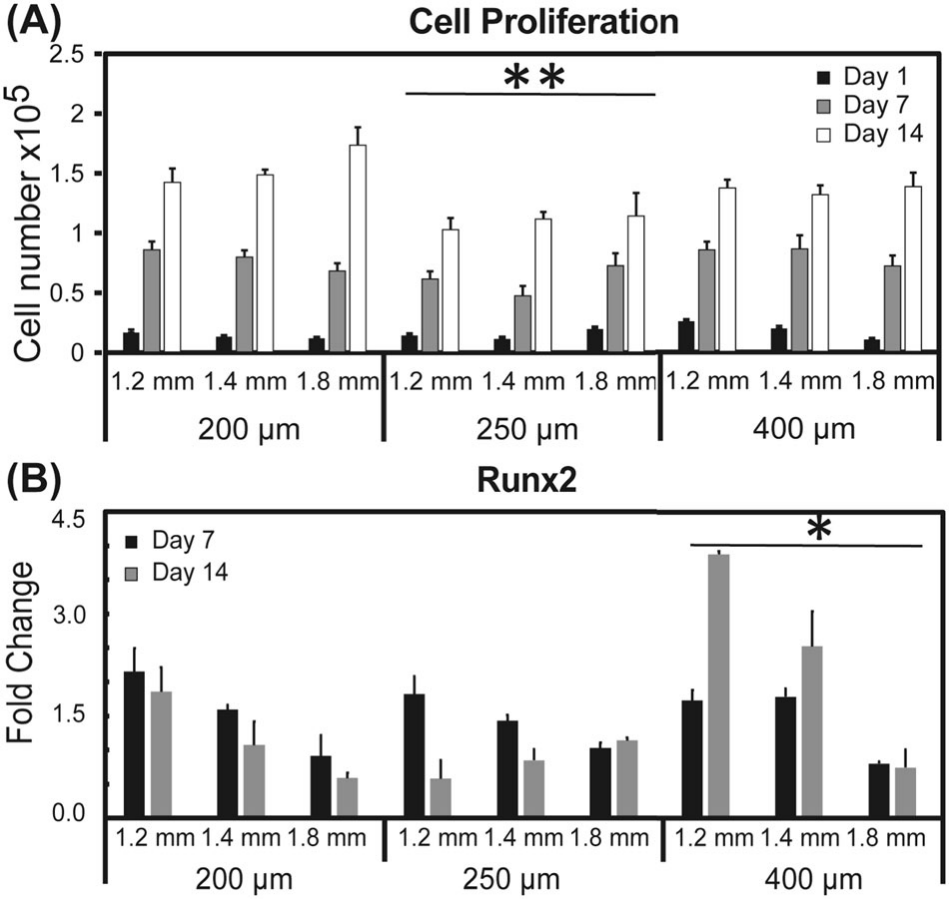
**A** Cell proliferation of MSCs on scaffolds printed with varying inner spacings and tip diameters. All groups were significantly different (*p* < 0.001) across all time points (not shown). Scaffolds printed using a tip diameter of 250 µm yielded cell proliferation that was significantly (**p < 0.001) than scaffolds printed with the other tip diameters. **B** *Runx2* expression of MSCs on scaffolds printed with varying inner spacing and tip diameters. Gene expression was significantly different (*p* < 0.05) between all inner spacings (not shown). *Runx2* expression of cells seeded on scaffolds printed with a 400 µm tip diameter were significantly higher (*p < 0.05) than those scaffolds printed with other tip diameters

There was a significant (*p* < 0.001) increase in cell proliferation across all timepoints (Fig. 9A). Cell proliferation from scaffolds printed with a 250 µm tip diameter were significantly (*p* < 0.001) different when compared to the other tip diameters (Fig. 9A). There were significant differences (*p* < 0.05) in Runx2 expression across print spacing (not shown) with Runx2 expression on scaffolds printed with a tip diameter of 400 µm different (*p* < 0.05) than tip diameters of 200 or 250 µm (Fig. 9B).

## Discussion

The primary objectives of the present study were (1) to develop β-TCP-based bioinks which maximized ceramic content while ensuring handleability (in order to print the green body) and post-sintering structural stability and (2) to evaluate the mechanical strength of the porous scaffold architectures printed using the optimized bioink formulation. A ceramic-based ink for direct writing or robocasting-type 3D printing (designed to print macroporous architectures without additional support material) has to meet two primary criteria: (1) suitable binders and high ceramic content (to ensure grain fusion during post-processing and sintering); and (2) ink solutions conducive to extrusion through a nozzle that either dries rapidly or has a phase transition near room temperature and also maintains sufficient cohesion to hold its shape immediately after deposition.

The novel bioink formulations designed, developed, and tested in the present study used gelatin and carboxymethyl cellulose (CMC) to meet the aforementioned objectives. Gelatin [28], formed by denaturing collagen at elevated temperatures, is frequently used to prepare hydrogels in aqueous solutions [28] to obtain injectable calcium phosphate cements for orthopedic applications [29–32]. Depending on the aqueous dilution, gelatin has a phase transition from sol-to-gel states between 30 and 40 °C [29]; in the present study heating the ink to 37 °C enabled 3D printing in a continuous manner when the ceramic fraction of the ink was greater than 50% (Table 1). Additionally, during the ink formulation, a final liquid-to-powder ratio of 0.45 was chosen because it enabled rapid thermosetting of the bioink, without interrupting the printing process between layers, and prevented settling of the bioink on the printing tip during holding intervals, thus avoiding clogging of the nozzle.

A range of CMC and other cellulose-based additives have been used as either binders or gelling agents to maintain cohesion in calcium phosphate formulations [33, 34]. In the present study, addition of 1–3% CMC resulted in overall increase of the β-TCP fraction, while maintaining ink consistency for continuous 3D printing; furthermore, incorporation of 3% CMC proved optimal to maintain ink cohesion. In comparison, the reported much lower 1% CMC volume fraction [35], used to prepare calcium phosphate slurries for template coating procedures, was preferred in order to maintain a greater sol-like consistency to obtain a dispersed, yet continuous coating. Furthermore, use of both gelatin and CMC in the present study, resulted in increased ß-TCP fraction (75%) in the ink (Fig. 1). In comparison, ceramic fractions in the 35–55% range were reported in direct-ink writing and robocasting methods, and were as high as 60% in fused deposition modeling methods reported in the literature [13]. The effect of 75% ß-TCP fraction on the mechanical properties of the bioinks tested in the present study was most evident in the significantly increased elastic modulus (*p* < 0.05) and ultimate strength (*p* < 0.05) under compression of the tested 3D-printed scaffolds (Fig. 3). We have successfully developed a bioink that allows for continuous printing, using a standard commercial 3D pneumatic printer, that requires no special curing process and, post-sintering, results in a scaffold that contains intergranular porosity in addition to the designed macropores while still retaining reasonable mechanical properties for bone tissue engineering applications.

Another contribution of the present study was optimization of the bioink and scaffold architectural parameters to simultaneously achieve both increased porosity and increased mechanical strength. It is established that the mechanical properties of sintered materials correlate closely with grain size [36]. As reported in the literature, β-TCP scaffolds prepared using robocasting methods and sintering to 1300 °C, resulted in final grain sizes of 7.4 µm with scaffold strengths between 10 and 15 MPa and a total porosity of 40% [8]. Another study using compacted pure β-TCP, which was densified at 1250 °C, reported a final grain size of 4.6 µm and compact material strength of ~75 MPa [37]. Moreover, β-TCP and phosphate-based glass composite scaffolds (prepared by extrusion spheronization and then sintered at 1100 °C), resulted in both hexagonal and tetragonal grains ranging from 1 to 20 µm in size; compressive strength of 14 MPa was reported for such scaffolds when the total porosity was 48% [38]. In comparison, by increasing the β-TCP fraction in the ink, and by sintering at 1240 °C, hexagonal grain sizes of 6.3 µm were obtained in the present study (Fig. 1b). The mechanical properties were optimized to achieve a compressive strength of 13.6 MPa (Fig. 6a) for scaffolds that were 53% porous (Fig. 8). In comparison to results reported when other 3D printing approaches for biomaterial scaffolds were used, a mesoporous, bioglass-poly vinyl alcohol, 3D-printed ceramic exhibited compressive strength of 16 MPa at 60% porosity [39]; a compressive strength of 5.5–6.6 MPa was achieved with 3D-printed β-TCP at 51–54% porosity [2, 40]. These findings demonstrate that the 75% TCP + CMC ink formulation developed in the current study can be used for 3D plotting/direct writing applications and then produce sintered macroporous β-TCP scaffolds with comparable, if not superior, mechanical properties compared to results obtained using other available manufacturing techniques.

Methods ranging from freeze-casting, to foam replication, particle leaching, or alternative additive manufacturing methods such as photopolymerization, direct light processing, fused deposition modeling, and powder binder jetting have all been used to create 3D calcium phosphate scaffolds (reviewed and summarized in [1, 25]). The porosity of structures typically falls in one of three bands depending on process parameters: <40%, 50–60%, and >80% with resultant mechanical strength ranging from >50 MPa, to 5–20 MPa and ~2 MPa, respectively. In the current study, the scaffolds generated had macroporosity of 50–70% (Fig. 7) and compressive strength of 10–15 MPa (Fig. 8) which is relatively high for other comparable architecture-strength combinations reported. One possible explanation for this is that the primary failure mode of the open cellular brittle foams created by the 3D printing process is closely related to the flexure and successive buckling fracture of the individual struts, and thus the elastic modulus and compressive strength are both proportional to exponents of the solid volume fraction of the printed scaffolds and hence proportional to exponents of (tip diameter/print spacing) following the model proposed by Gibson and Ashby [41]. This is further supported by the data in the current study where the ultimate compressive strength of the porous foams (Fig. 8) is very similar to the brittle flexural strength of a pure sintered beam of the same bioink (Fig. 3). This further suggests that polymer coating or infiltration methods are likely to have a significant impact on improving the mechanical properties of the scaffolds as reported for calcium phosphate scaffolds manufactured from other techniques.

While high strength and high porosity are essential for various applications of microporous ceramics, biomedical-related applications have unique additional requirements regarding scaffold architecture. Specifically, for tissue engineering applications, highly porous scaffolds are required because they provide appropriate micro-structures and environmental cues as well as mechanical support to promote tissue regeneration [5, 6]. The open porosity and pore organization of such scaffolds determines available surface area (which promotes cell attachment), as well as scaffold permeability (which controls oxygen, nutrient and cell-metabolic waste transport) [42]. At the present time, there is no clear consensus on either optimal pore size or pore configuration for ideal bone tissue engineering scaffolds. While implanted scaffolds with minimum pore size of 100 µm supported in-growth of only bone tissue in vivo, scaffolds with a pore size of 500 µm sustained in-growth of both organized bone tissue and new blood vessels [43]. Moreover, scaffold pore size affects functions at both the cellular- and molecular levels. For example, in an in vitro study investigating differentiation of mesenchymal stem cells cultured on 200 µm pore size scaffolds reported enhanced osteogenenic differentiation in comparison to cells cultured on 500 µm pore size calcium phosphate scaffolds [44]. An explanation for these observations is that scaffolds with smaller pore sizes exhibit an increased available surface area. The resultant biological effects stem from increased pre-adsorption of select proteins [45] that modulate consequent cell attachment [46] on scaffold surfaces. Pore size, porosity, and surface area are all interdependent architectural variables in scaffold design. For example, surface curvature, which is greater for smaller pore sizes at the same porosity, provides increased surface area pertinent to adhesion and thus survival of anchorage-dependent cells, as well as dictates how bone cells organize and function to produce extracellular matrix [47, 48]. From the in vitro analysis in the current study, scaffolds composed of 75% TCP + CMC a support cell attachment and early osteogenic commitment. As cell proliferation is determined by the surface area available, varying the tip diameter allows for control of the level of cell proliferation of 3D-printed scaffolds (Fig. 9). Additionally, pore size influences the level of differentiation, which is affected by the inner line spacing of scaffolds. The macropore channel diameters of the scaffolds prepared in the present study ranged between 800 and 1600 µm (Fig. 5); after the 35% sintering shrinkage, the mean pore diameters observed for the scaffolds tested ranged between 250 and 700 µm (Fig. 7f). These pore sizes span the 250 to >500 µm range suggested in the literature [9, 35, 43, 49] to support both new blood vessel and bone tissue in-growth within scaffolds implanted in vivo.

Porous architectures are necessary for successful tissue engineering applications because they are the channels for biological fluid conductance [50]. The importance of this requirement cannot be overstated because survival first, and subsequent functions of cells pertinent to new tissue formation, critically depends on oxygen, nutrient and cell-metabolic waste transport [51]. Permeability, a measure of fluid transport through porous media, quantitatively describes a scaffold property independent of scaffold size and fluid used. In this respect, it was reported that (1) porosity, (2) pore size and distribution, (3) pore interconnectivity or tortuosity, (4) fenestration size and distribution, and (5) pore orientation, all affect scaffold permeability, emphasizing the strong correlation with scaffold architecture [52, 53]. In the present study, the permeability of the scaffold architectures tested ranged from 1.77–11.3 × 10^−10^ m^2^. This result is comparable to the 1.2–80.5 × 10^−10^ m^2^ [54–56] range of permeability of human trabecular bone in various anatomical locations as well as to the permeability of coralline hydroxyapatite (specifically, 5 × 10^−10^ m^2^) [57] and calcium phosphate scaffolds prepared by template coating (specifically, 4.8 × 10^−10^ m^2^) [42]. Studies of animal models addressed the impact of scaffold fluid conductance on both new bone tissue formation and new blood vessel infiltration, a crucial requirement for survival and function of vascularized tissues [50]. A minimum threshold fluid conductance of 1.5 × 10^−10^ m^3^s^−1^Pa^−1^ was reported as necessary to achieve vascularized tissue formation within scaffolds [50]. Since, in the present study, the conductance of the β-TCP scaffolds (specifically, 4.82–31.19 × 10^−10^ m^3^s^−1^Pa^−1^) was well above the aforementioned threshold values (Fig. 6b), these scaffolds have the potential to promote, and sustain, new bone formation and regeneration in vivo.

Multiple techniques reported in the literature have attempted to enhance the mechanical properties as well as the bioactivity of β-TCP-based materials, including doping with other ceramics (such as magnesia, zinc oxide, and silica) [37] and infiltration with various polymer solutions [58, 59]. The approach of 3D printing by direct-ink writing and then sintering the resultant scaffolds designed, developed, and tested in the present study lends itself well to introducing dopants either during the preparation of the bioink or during the printing process. Since the sintered β-TCP scaffolds produced in the present study exhibited intergranular microporosity (Fig. 2), they are also suitable for introducing polymer infiltration after sintering to further improve the mechanical strength and provide resistance to brittle fracture. Furthermore, since macroporous β-TCP scaffolds are excellent delivery platforms for growth factors [60] and antibiotics [61], they are ideally suited for several other biomedical applications [12].

## Conclusion

The present study demonstrated that novel bioink formulations with a high β-TCP (75%) to gelatin (25%) ratio were stabilized by the addition of 3% CMC for successful 3D printing of macroporous scaffolds. During printing, the novel formulations had a cohesive structure, and after post-processing sintering, the printed scaffolds retained their shape and exhibited uniform volumetric shrinkage. The final 3D architectures retained macroporosity and also included microporosity within the sintered ceramic. Varying the print nozzle thickness and the pore spacing enabled printed scaffold architectures with a range of porosities and strengths. The scaffolds prepared via direct 3D printing and sintering achieved compressive strengths comparable, if not superior, to β-TCP scaffolds with similar porosities produced using other reported manufacturing techniques. In summary, the 3D printing using high content β-TCP ink enables production of porous, structurally stable scaffolds suitable for multiple applications including biomedical ones in bone and craniomaxillofacial tissue engineering.

## Supplementary information

Supplementary Information

Supplementary Data

## References

[1] Guda T, Appleford M, Oh S, Ong JL (2008). A cellular perspective to bioceramic scaffolds for bone tissue engineering: the state of the art. Curr Top Med Chem.

[2] Tarafder S, Balla VK, Davies NM, Bandyopadhyay A, Bose S (2013). Microwave-sintered 3D printed tricalcium phosphate scaffolds for bone tissue engineering. J Tissue Eng Regen Med.

[3] Kang Y, Scully A, Young DA, Kim S, Tsao H, Sen M (2011). Enhanced mechanical performance and biological evaluation of a PLGA coated β-TCP composite scaffold for load-bearing applications. Eur Polym J.

[4] Bose S, Vahabzadeh S, Bandyopadhyay A (2013). Bone tissue engineering using 3D printing. Mater Today.

[5] Hollister SJ (2005). Porous scaffold design for tissue engineering. Nat Mater.

[6] Hutmacher DW (2001). Scaffold design and fabrication technologies for engineering tissues—state of the art and future perspectives. J Biomater Sci Polym Ed.

[7] Studart AR, Gonzenbach UT, Tervoort E, Gauckler LJ (2006). Processing routes to macroporous ceramics: a review. J Am Ceram Soc.

[8] Miranda P, Saiz E, Gryn K, Tomsia AP (2006). Sintering and robocasting of β-tricalcium phosphate scaffolds for orthopaedic applications. Acta Biomater.

[9] Guda T, Walker JA, Singleton B, Hernandez J, Oh DS, Appleford MR (2014). Hydroxyapatite scaffold pore architecture effects in large bone defects in vivo. J Biomater Appl.

[10] Jongpaiboonkit L, Halloran JW, Hollister SJ (2006). Internal structure evaluation of three-dimensional calcium phosphate bone scaffolds: a micro-computed tomographic study. J Am Ceram Soc.

[11] Lewis JA, Smay JE, Stuecker J, Cesarano J (2006). Direct ink writing of three-dimensional ceramic structures. J Am Ceram Soc.

[12] Trombetta R, Inzana JA, Schwarz EM, Kates SL, Awad HA (2017). 3D printing of calcium phosphate ceramics for bone tissue engineering and drug delivery. Ann Biomed Eng.

[13] Zocca A, Colombo P, Gomes CM, Günster J (2015). Additive manufacturing of ceramics: issues, potentialities, and opportunities. J Am Ceram Soc.

[14] de Vasconcellos LMR, Camporês KL, de Alcântara Abdala JM, Vieira MN, de Oliveira IR (2020). Biological and microbiological behavior of calcium aluminate cement-based blend for filling of bone defects. J Mater Sci Mater Med.

[15] Deville S, Saiz E, Tomsia AP (2006). Freeze casting of hydroxyapatite scaffolds for bone tissue engineering. Biomaterials..

[16] Macchetta A, Turner IG, Bowen CR (2009). Fabrication of HA/TCP scaffolds with a graded and porous structure using a camphene-based freeze-casting method. Acta Biomater.

[17] Chang H-K, Chen P-Y (2020). Synthesis of silica-based scaffolds with high porosity and controllable microstructure by a sintering-free sol–gel/freeze-casting hybrid method under mild conditions. J Mater Res Technol.

[18] Sadowska JM, Wei F, Guo J, Guillem-Marti J, Lin Z, Ginebra M-P (2019). The effect of biomimetic calcium deficient hydroxyapatite and sintered β-tricalcium phosphate on osteoimmune reaction and osteogenesis. Acta Biomater.

[19] Yu L, Rowe DW, Perera IP, Zhang J, Suib SL, Xin X (2020). Intrafibrillar mineralized collagen–hydroxyapatite-based scaffolds for bone regeneration. ACS Appl Mater Interfaces.

[20] Maleki-Ghaleh H, Khalil-Allafi J, Keikhosravani P, Etminanfar M, Behnamian Y (2020). Effect of nano-zirconia on microstructure and biological behavior of hydroxyapatite-based bone scaffolds. J Mater Eng Perfor.

[21] Song X, Tetik H, Jirakittsonthon T, Parandoush P, Yang G, Lee D (2019). Biomimetic 3D printing of hierarchical and interconnected porous hydroxyapatite structures with high mechanical strength for bone cell culture. Adv Eng Mater.

[22] Hayashi K, Kato N, Kato M, Ishikawa K (2021). Impacts of channel direction on bone tissue engineering in 3D-printed carbonate apatite scaffolds. Mater Des.

[23] Baino F, Magnaterra G, Fiume E, Schiavi A, Tofan L-P, Schwentenwein M, et al. DLP stereolithography of hydroxyapatite scaffolds with bone-like architecture, permeability and mechanical properties. J Am Ceram Soc. 2021. 10.1111/jace.17843.

[24] Chai W, Wei Q, Yang M, Ji K, Guo Y, Wei S (2020). The printability of three water based polymeric binders and their effects on the properties of 3D printed hydroxyapatite bone scaffold. Ceram Int.

[25] Li X, Yuan Y, Liu L, Leung Y-S, Chen Y, Guo Y (2020). 3D printing of hydroxyapatite/tricalcium phosphate scaffold with hierarchical porous structure for bone regeneration. Bio-Des Manuf.

[26] Shao H, He J, Lin T, Zhang Z, Zhang Y, Liu S (2019). 3D gel-printing of hydroxyapatite scaffold for bone tissue engineering. Ceram Int.

[27] Zhou T, Zhang L, Yao Q, Ma Y, Hou C, Sun B (2020). SLA 3D printing of high quality spine shaped β-TCP bioceramics for the hard tissue repair applications. Ceram Int.

[28] Prystupa DA, Donald AM (1996). Infrared study of gelatin conformations in the gel and sol states. Polymer Gels Networks.

[29] Bigi A, Bracci B, Panzavolta S (2004). Effect of added gelatin on the properties of calcium phosphate cement. Biomaterials.

[30] Bigi A, Torricelli P, Fini M, Bracci B, Panzavolta S, Sturba L (2004). A biomimetic gelatin-calcium phosphate bone cement. Int J Artif Organs.

[31] Habraken WJEM, de Jonge LT, Wolke JGC, Yubao L, Mikos AG, Jansen JA (2008). Introduction of gelatin microspheres into an injectable calcium phosphate cement. J Biomed Mater Res Part A.

[32] Shie M-Y, Chen DC-H, Wang C-Y, Chiang T-Y, Ding S-J (2008). Immersion behavior of gelatin-containing calcium phosphate cement. Acta Biomater.

[33] Kobayashi H, Fujishiro T, Belkoff SM, Kobayashi N, Turner AS, Seim HB (2009). Long‐term evaluation of a calcium phosphate bone cement with carboxymethyl cellulose in a vertebral defect model. J Biomed Mater Res Part A.

[34] Perez RA, Kim H-W, Ginebra M-P (2012). Polymeric additives to enhance the functional properties of calcium phosphate cements. J Tissue Eng.

[35] Guda T, Walker JA, Singleton BM, Hernandez JW, Son JS, Kim SG (2013). Guided bone regeneration in long-bone defects with a structural hydroxyapatite graft and collagen membrane. Tissue Eng Part A.

[36] Wang J, Shaw LL (2009). Nanocrystalline hydroxyapatite with simultaneous enhancements in hardness and toughness. Biomaterials.

[37] Bandyopadhyay A, Bernard S, Xue W, Bose S (2006). Calcium phosphate-based resorbable ceramics: influence of MgO, ZnO, and SiO2 Dopants. J Am Ceram Soc.

[38] He F, Tian Y, Fang X, Lu T, Li J, Shi X (2018). Fabrication of β-tricalcium phosphate composite ceramic scaffolds based on spheres prepared by extrusion-spheronization. J Am Ceram Soc.

[39] Wu C, Luo Y, Cuniberti G, Xiao Y, Gelinsky M (2011). Three-dimensional printing of hierarchical and tough mesoporous bioactive glass scaffolds with a controllable pore architecture, excellent mechanical strength and mineralization ability. Acta Biomater.

[40] Fielding GA, Bandyopadhyay A, Bose S (2012). Effects of silica and zinc oxide doping on mechanical and biological properties of 3D printed tricalcium phosphate tissue engineering scaffolds. Dent Mater.

[41] Gibson LJ, Ashby M. The mechanics of foams: basic results. In: Gibson LJ, Ashby M, editors. Cellular solids: structure and properties. 2nd ed. Cambridge University Press, Cambridge; 1997. p. 175–231.

[42] Guda T, Oh S, Appleford MR, Ong JL (2012). Bilayer hydroxyapatite scaffolds for maxillofacial bone tissue engineering. Int J Oral Maxillofac Implants.

[43] Tsiridis E, Gurav N, Bailey G, Sambrook R, Di Silvio L (2006). A novel ex vivo culture system for studying bone repair. Injury..

[44] Mygind T, Stiehler M, Baatrup A, Li H, Zou X, Flyvbjerg A (2007). Mesenchymal stem cell in growth and differentiation on coralline hydroxyapatite scaffolds. Biomaterials..

[45] Puleo DA, Bizios R (1992). Mechanisms of fibronectin-mediated attachment of osteoblasts to substrates in vitro. Bone Mineral.

[46] Malik MA, Puleo DA, Bizios R, Doremus RH (1992). Osteoblasts on hydroxyapatite, alumina and bone surfaces in vitro; morphology during the first 2 h of attachment. Biomaterials.

[47] Pilia M, Guda T, Pollot BE, Aguero V, Appleford MR (2014). Local microarchitecture affects mechanical properties of deposited extracellular matrix for osteonal regeneration. Mater Sci Eng C Mater Biol Appl.

[48] Pilia M, Guda T, Shiels SM, Appleford MR (2013). Influence of substrate curvature on osteoblast orientation and extracellular matrix deposition. J Biol Eng.

[49] Guda T, Walker JA, Pollot BE, Appleford MR, Oh S, Ong JL (2011). In vivo performance of bilayer hydroxyapatite scaffolds for bone tissue regeneration in the rabbit radius. J Mater Sci Mater Med.

[50] Hui PW, Leung PC, Sher A (1996). Fluid conductance of cancellous bone graft as a predictor for graft-host interface healing. J Biomech.

[51] O’Brien FJ, Harley BA, Waller MA, Yannas IV, Gibson LJ, Prendergast PJ (2007). The effect of pore size on permeability and cell attachment in collagen scaffolds for tissue engineering. Technol Health Care.

[52] Arramon Y, Nauman E. The intrinsic permeability of cancellous bone. In: Cowin SC, editor. Bone mechanics handbook. 2nd ed. Boca Raton: CRC Press; 2001. p. 251–317.

[53] Li S, De Wijn JR, Li J, Layrolle P, De Groot K (2003). Macroporous biphasic calcium phosphate scaffold with high permeability/porosity ratio. Tissue Eng.

[54] Beaudoin AJ, Mihalko WM, Krause WR (1991). Finite element modelling of polymethylmethacrylate flow through cancellous bone. J Biomech.

[55] Grimm MJ, Williams JL (1997). Measurements of permeability in human calcaneal trabecular bone. J Biomech.

[56] Nauman EA, Fong KE, Keaveny TM (1999). Dependence of intertrabecular permeability on flow direction and anatomic site. Ann Biomed Eng.

[57] Haddock SM, Debes JC, Nauman EA, Fong KE, Arramon YP, Keaveny TM (1999). Structure-function relationships for coralline hydroxyapatite bone substitute. J Biomed Mater Res.

[58] Cornelsen M, Petersen S, Dietsch K, Rudolph A, Schmitz K, Sternberg K, et al. Infiltration of 3D printed tricalciumphosphate scaffolds with biodegradable polymers and biomolecules for local drug delivery. Biomedizinische Technik. 2013;58. 10.1515/bmt-2013-4090. 10.1515/bmt-2013-4090 24042685

[59] Martínez-Vázquez FJ, Perera FH, Miranda P, Pajares A, Guiberteau F (2010). Improving the compressive strength of bioceramic robocast scaffolds by polymer infiltration. Acta Biomater.

[60] Shiels S, Oh S, Bae C, Guda T, Singleton B, Dean DD (2012). Evaluation of BMP-2 tethered polyelectrolyte coatings on hydroxyapatite scaffolds in vivo. J Biomed Mater Res B Appl Biomater.

[61] Pearson JJ, Gerken N, Bae C, Lee KB, Satsangi A, McBride S (2020). In vivo hydroxyapatite scaffold performance in infected bone defects. J Biomed Mater Res B Appl Biomater.

