# Supplementary material for: Development of bioinks for 3D printing microporous, sintered calcium phosphate scaffolds
Source: J Mater Sci Mater Med. 2021 Aug 14;32(8):94. doi: 10.1007/s10856-021-06569-9 (PMC8364524; doi:10.1007/s10856-021-06569-9)
Supplement: Supplementary file 1 — Supplementary Information [file 10856_2021_6569_MOESM1_ESM.docx]

**
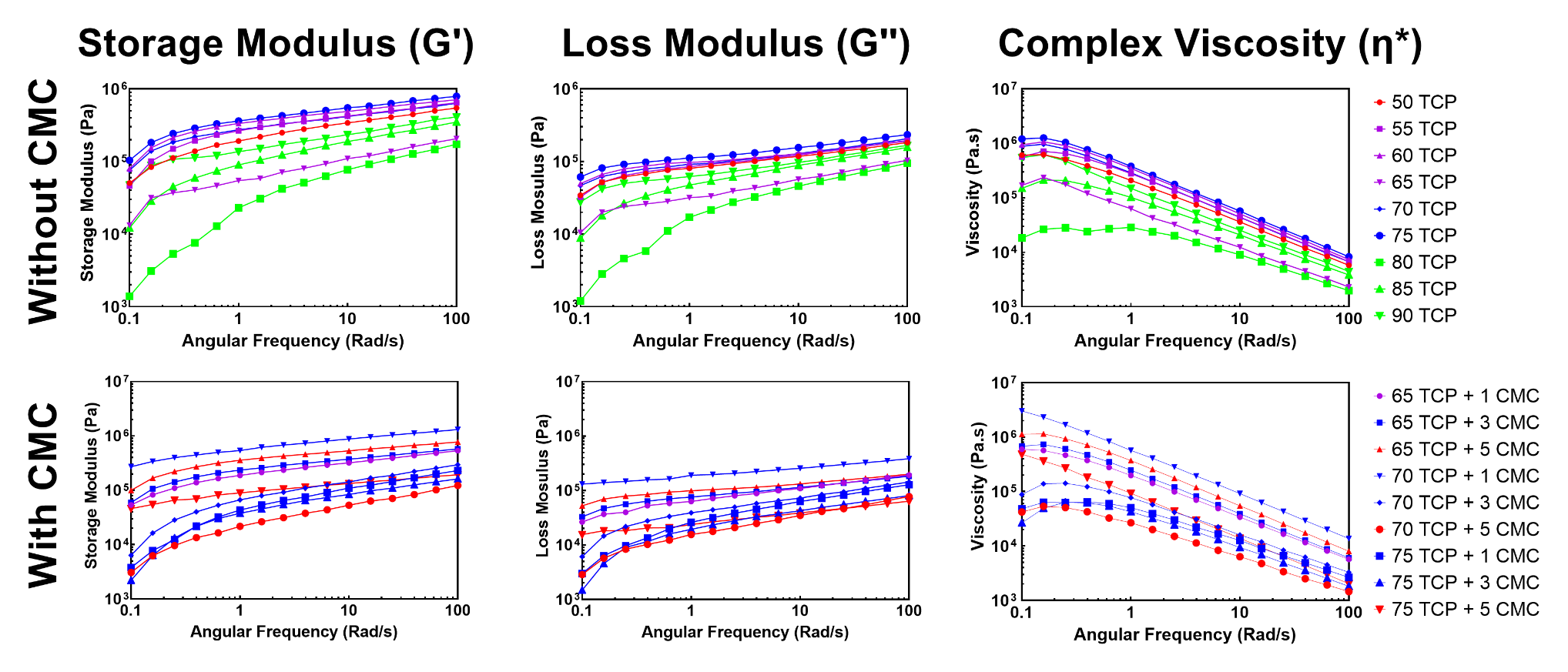
**

**Figure S1**. Rheological evaluation of the various inks tested in Table 1. The storage modulus, loss modulus and complex viscosity of the inks evaluated (top row) without CMC and (bottom row) with CMC included. Blue: printable, handleable and sinterable; Purple: printable, handleable, not sinterable; Green: printable, not handleable; Red: not printable

**Supplementary Data**

**Methods:** The rheological properties of the various inks listed in Table 1 were tested using an MCR 502 rheometer (Anton Paar, Graz, Austria) in 25mm parallel plate setup with a 1 mm gap. An amplitude sweep was first performed at constant 10 s^-1^ frequency to determine the linear visco-elastic region (LVER). All samples were equilibrated in a water bath set to 40^o^C for 1 hour prior to testing. The samples were transferred from the bath to the instrument within 5 minutes. Once samples were loaded in the machine, frequency sweeps were performed on all the samples from 0.1 to 100 rad/sec at a constant shear rate of 0.05%.

**Brief discussion:** Without CMC, the reason why certain inks do not print was because the storage modulus (G') was not higher than the loss modulus (G"), especially at low shear rates (such as experienced when the printing was carried out between 50 and 70 kPa). Only the inks with the highest complex viscocity were actually sinterable in shape. At low CMC concentrations (1% or 3%), the concern was not printability, but rather that the material showed low elasticity as a solid at low frequencies (angular velocity) when the TCP concentration was low (65%). At high CMC concentrations, the printability was affected because of extremely high cohesion during print.
